# A Symmetrical Quasi-Synchronous Step-Transition Folded Waveguide Slow Wave Structure for 650 GHz Traveling Wave Tubes

**DOI:** 10.3390/s24165289

**Published:** 2024-08-15

**Authors:** Duo Xu, Tenglong He, Yuan Zheng, Zhigang Lu, Huarong Gong, Zhanliang Wang, Zhaoyun Duan, Shaomeng Wang

**Affiliations:** 1 School of Electronic Science and Engineering, University of Electronic Science and Technology of China, No. 2006, Xiyuan Ave., West Hi-Tech Zone, Chengdu 611731, China; d.xu@uestc.edu.cn (D.X.); zyzheng@uestc.edu.cn (Y.Z.); lzhgchnn@uestc.edu.cn (Z.L.); hrgong@uestc.edu.cn (H.G.); wangzl@uestc.edu.cn (Z.W.); zhyduan@uestc.edu.cn (Z.D.); 2 Southwest Electronic Equipment Research Institute, No. 496, Yingkang West Road, Jinniu Distinct, Chengdu 610036, China; faithhill@foxmail.com

**Keywords:** self-oscillation suppressing, terahertz radiation, traveling wave tubes

## Abstract

For the purpose of improving performance and reducing the fabrication difficulty of terahertz traveling wave tubes (TWTs), this paper proposes a novel single-section high-gain slow wave structure (SWS), which is named the symmetrical quasi-synchronous step-transition (SQSST) folded waveguide (FW). The SQSST-FW SWS has an artificially designed quasi-synchronous region (QSR) to suppress self-oscillations for sustaining a high gain in an untruncated circuit. Simultaneously, a symmetrical design can improve the efficiency performance to some extent. A prototype of the SQSST-FW SWS for 650 GHz TWTs is designed based on small-signal analysis and numerical simulation. The simulation results indicate that the maximum saturation gain of the designed 650 GHz SQSST-FW TWT is 39.1 dB in a 34.3 mm slow wave circuit, occurring at the 645 GHz point when a 25.4 kV 15 mA electron beam and a 0.43 mW sinusoidal input signal are applied. In addition, a maximum output power exceeding 4 W is observed at the 648 GHz point using the same beam with an increased input power of around 2.8 mW.

## 1. Introduction

Being at the overlap of electronics and photonics, terahertz radiation has many attractive properties and has great potential for applications in areas such as wireless communications [1,2,3,4], imaging [5,6,7], and biomedicine [8,9,10]. On the other hand, the terahertz band is known as the “terahertz gap” due to the inadequate power of sources.

As a popular type of vacuum electronic device, the TWT can amplify terahertz signals and give remarkable output power with much higher efficiency over solid-state power amplifiers, which is critical for the application of terahertz waves.

In recent years, many institutes have conducted experimental research on terahertz TWTs. Hu et al. [11,12] reported the development of a 0.22 THz and a 0.34 THz TWT at the China Academy of Engineering Physics. Their maximum output powers reach 30 W and 3.17 W, respectively. The corresponding gains are 31.2 dB and 26.2 dB, respectively. Liu et al. [13] of the Aerospace Information Research Institute reported a power amplifying scheme by two cascaded TWTs at 0.22 THz and achieved a 60 W peak output power and a 33 dB peak gain. The recent advances in terahertz TWTs at the Beijing Vacuum Electronic Research Institute were reported by Pan et al. [14,15]; they successfully fabricated an 11.9 W 25.5 dB 0.26 THz TWT and a 1.6 W 0.34 THz power module, in which a TWT with a maximum gain of 22 dB was applied. Northrop Grumman Corporation developed a series of high-frequency terahertz TWTs. The operating frequency band includes 0.67 THz [16], 0.85 THz [17], and 1.03 THz [18]. Among them, the 1.03 THz TWT holds the highest operating frequency record for TWTs to date, which has a peak output power of 29 mW and a peak gain of 20 dB.

It is known that fabrication tolerance is very crucial at the terahertz band; thus, a SWS with a simple structure is helpful for the success of a terahertz TWT. In our previous work, we proposed an attenuator-free SWS, named the modified angular log-periodic (MALP) FW [19], for single-section high-gain terahertz TWTs and validated its principle by a prototype TWT at the Ka band [20]. Based on the concept of the QSR in the MALP-FW SWS, this paper proposed a novel SQSST-FW SWS to further reduce the fabrication difficulty and improve the output power of terahertz TWTs. In addition, a prototype 650 GHz SQSST-FW TWT is designed in this paper as an example to illuminate the scheme and design method. According to the simulation, it can produce an output of over 3 W with an input of 0.4 mW around 650 GHz. As a high-power terahertz source, the SQSST-FW TWT would benefit the development of the terahertz sensors for fields including nondestructive inspection and testing, electromagnetic biology effects, and recognition of protein structural states.

The structure of this paper is as follows: Section 2 describes the scheme of the SQSST-FW SWS; Section 3 briefly reviews the small-signal theoretical foundation of the backward-wave oscillations in the SQSST-FW SWS; Section 4 verifies the accuracy of the small-signal theory in predicting the starting length of oscillation (hereafter referred to as starting length) by using particle-in-cell (PIC) simulations; Section 5 introduces the simulation for the performance of the designed 650 GHz TWT.

## 2. Scheme of SQSST-FW SWS

**Figure 1:**
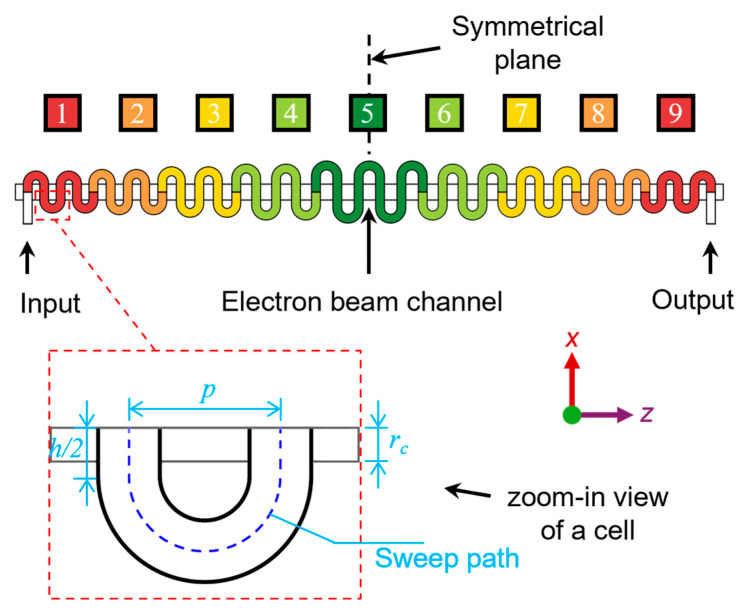
A schematic diagram of the SQSST-FW SWS.

Figure 1 shows the overall structure of the SQSST-FW SWS, including 9 segments marked in different colors. The overall structure is symmetrical about the middle plane in the *z*-direction. The zoom-in view shows the line art of a unit cell, where the deep blue dash curve is the meandering path. The total length and the projected length in the *z*-axis of the meandering path are *L* and *p*, respectively. Within one segment, there are several unit cells with the same *L* and *p*, which are different in different segments.

The first half of the SQSST-FW SWS becomes wider gradually, being similar to that in [19]. By changing the values of *h* and *p*, we made the SQSST-FW SWS have a QSR. The significant difference from the MALP-FW SWS is that the SQSST-FW SWS is step-transition by segments, while the latter is transition by every cell. The novel topology reduces the requirement for SWS machining accuracy. On the other hand, the performance of suppressing self-oscillations is also reduced. Hence, the number of cells in every segment should be limited to avoid oscillations, which will be discussed in Section 3 and Section 4.

In addition, the symmetrical design makes the axial wave phase velocities vary in reversed trends in the two halves, allowing for the creation of a positive-/negative-tapering phase-velocity curve in a certain frequency range and the improvement of output power.

With waveguide cross-sectional dimensions of 0.26 mm × 0.055 mm and the electron beam channel radius *r_c_* of 0.05 mm, Table 1 lists the other dimensions of the cells in different segments of the 650 GHz SQSST-FW SWS, where the subscript represents the number of the segment.

As mentioned above, the number of cells in every segment should be limited due to the reduction in the oscillation-suppressing performance. A simple method to determine this number is based on the small-signal theory introduced in the following sections.

## 3. Analysis of the Oscillations

The most common self-oscillations in TWTs can be divided into reflection oscillation and backward-wave oscillation [21]. The reflections of the wave are the main contributor to the reflection oscillations because they construct the energy feedback loop. Fortunately, the oscillations caused by the reflections can be naturally suppressed in the terahertz TWTs due to the high transmission loss. Therefore, this section focuses on the analysis of backward-wave oscillations.

The small-signal equations of backward-wave oscillators (BWOs), whose detailed derivation was given in Liu’s book [21], were employed to evaluate the effect of backward-wave oscillations in the TWT. The key equations are reorganized and summarized here.

### 3.1. 1-D Characteristic Equation for BWOs

In the small-signal theory, one-dimensional (1-D) electronic Equation (1), which describes the effect of the circuit field on the electron beam, is the same for a BWO and a TWT.

(1) $$i_{1}=\frac{j\beta_{e}}{{\left(\Gamma -j\beta_{e}\right)}^{2}+\beta_{q}^{2}}\frac{I_{0}E_{c}}{2V_{0}}$$

Here, *i*_1_ is the AC component of the beam current; *β_e_* is the phase constant for the electron beam; Γ is the propagation constant of the waves with the beam loaded (to be solved); *β_q_* is the phase constant of the plasma wave; *I*_0_ is the dc beam current; *E_c_* is the circuit field; and *V*_0_ is the dc beam voltage.

The circuit equation, which describes the effect of the electron beam on the circuit field, for BWOs is also essentially the same as that for TWTs except for the sign change caused by the change in the equation of the definition of the interaction impedance, as follows:

(2) $$E_{c}=\frac{\Gamma^{2}\Gamma_{0}K_{c}}{\Gamma^{2}-\Gamma_{0}^{2}}i_{1}$$

where Γ_0_ is the propagation constant of the wave in the SWS without the electron beam loaded; *K_c_* is the interaction impedance.

Joining (1) and (2), the characteristic equation for BWOs can be obtained, as follows:

(3) $$\left(\frac{\Gamma^{2}\Gamma_{0}K_{c}}{\Gamma^{2}-\Gamma_{0}^{2}}\right)\left[\frac{j\beta_{e}}{{\left(\Gamma -j\beta_{e}\right)}^{2}+\beta_{q}^{2}}\frac{I_{0}}{2V_{0}}\right]-1=0$$

Following the method for solving the characteristic equation for TWTs, let

(4) $$\left\{\begin{array}{c} \Gamma =j\beta_{e}-\beta_{e}C\delta \\ \Gamma_{0}=j\beta_{e}+j\beta_{e}Cb-\beta_{e}Cd \end{array}\right.$$

and

(5) δ=x+jy.

where *C*, *b*, and *d* are the gain, velocity, and loss parameters, respectively.

By substituting (4) and (5) into (3) and assuming that *C* << 1, and that *b*, *d*, and *δ* are not far from 1, one can obtain a pair of numerically solvable equations of *x* and *y*, as follows:

(6) $$\left\{\begin{array}{c} \left(x^{2}-y^{2}+4QC\right)\left(d-x\right)+2xy\left(y+b\right)=0\\ \left(x^{2}-y^{2}+4QC\right)\left(b+y\right)+2xy\left(x-d\right)-1=0 \end{array}\right.$$

### 3.2. The Starting Length of the Backward-Wave Oscillation

Similar to the case in TWTs, the gain equation for BWOs can be obtained by associating the boundary conditions, the electronic equation, and the current continuity equation. The gain of the backward wave “*G*” is

(7) $$G=\frac{\sum_{n=1}^{3}\left(\delta_{n+2}-\delta_{n+1}\right)\left(\delta_{n}^{2}+4QC\right)e^{j2\pi N}}{\sum_{n=1}^{3}\left(\delta_{n+2}-\delta_{n+1}\right)\left(\delta_{n}^{2}+4QC\right)e^{j2\pi CN\delta_{n}}}.$$

where *δ*_5_ = *δ*_2_; *δ*_4_ = *δ*_1_; *QC* is the space charge parameter; and *N* is the ratio of the circuit length to the wavelength for the electron beam.

Apparently, the starting condition of the backward-wave oscillation is *G* → ∞ or 1/*G* = 0, that is,

(8) $$\sum_{n=1}^{3}\frac{\left(\delta_{n}^{2}+4QC\right)e^{j2\pi CN\delta_{n}}}{\left(\delta_{n+2}-\delta_{n}\right)\left(\delta_{n+1}-\delta_{n}\right)}=0.$$

*δ*, *Q,* and *C* are all functions of the cell dimensions and the beam parameters, so the starting length of the backward-wave oscillation can be obtained by finding the proper *b* and *CN* that satisfy (8).

## 4. Starting Length in a Periodic SWS

### 4.1. Illumination of the Sample SWS

Here, a periodic FW SWS model was built and simulated for Segment 1 using PIC methods in CST [22] Particle Studio to verify the accuracy of the predicted starting length from (8).

**Figure 2:**
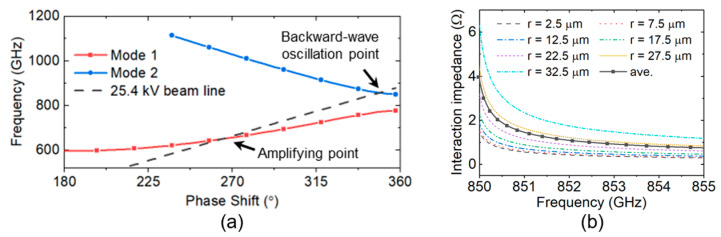
(**a**) A dispersion diagram and (**b**) the interaction impedance around the backward-wave oscillation point of the sample for periodic FW SWS.

The simulation results of the dispersion characteristic curves of this periodic SWS are shown in Figure 2a, from which the frequency of the backward-wave oscillation point is found to be around 850 GHz, and the interaction impedance around the backward-wave oscillation point is shown by Figure 2b, where *r* represents the radial coordinate.

To obtain the accurate oscillation frequency, a zero-drive PIC simulation model with an electron beam of 25.4 kV and 15 mA was built, and the simulation results found it to be 851 GHz, where the beam radius is 0.035 mm, and a 1 T uniform focusing magnetic field is applied. The average beam–wave interaction impedance of the backward wave at that point obtained by numerical simulation was 1.5 Ω.

### 4.2. Numerical Solutions of the Characteristic Equation

**Figure 3:**
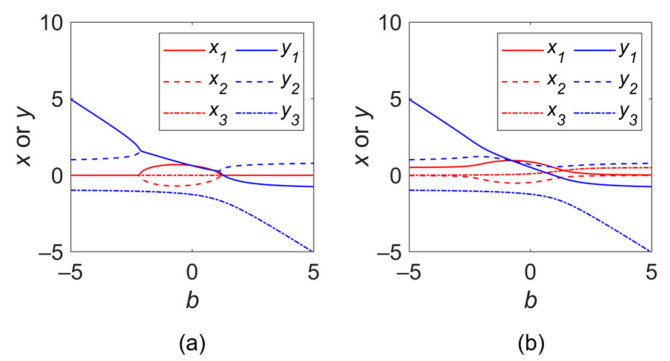
Solutions of *δ*: (**a**) *d* = 0; and (**b**) *d* = 0.507.

A prerequisite of numerically solving (6) is that *Q*, *C*, *b*, and *d* are known, where *Q* and *C* can be directly obtained by the known beam parameter and the interaction impedance. The loss parameter *d* is not directly calculable but can be obtained by substituting the simulation result of insertion loss into its definition equation. When neglecting the conductor loss of the SWS material, *d* is equal to zero apparently, while it changes to 0.507 if a conductivity of 2 × 10^7^ S/m was considered. The numerical solutions of (6) under these two conditions are given in Figure 3.

### 4.3. Calculated Starting Length Using the Small-Signal Equation

**Figure 4:**
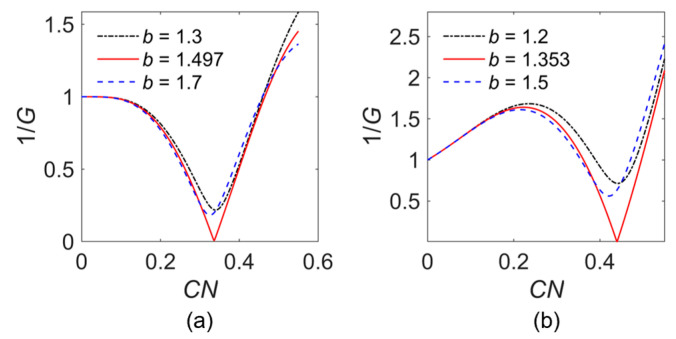
Variation of 1/*G* with *CN*: (**a**) *d* = 0; and (**b**) *d* = 0.507.

Once *δ* is solved, *G* or 1/*G* would be a singular value function of *CN*. The variation of 1/*G* with *CN* for different values of *b* is shown in Figure 4.

The red solid curve in Figure 4a indicates that the starting condition of the backward-wave oscillation in the loss-free model of the sample SWS is *b* = 1.497 and *CN* > 0.336, which corresponds to an actual starting length of about 5.95 mm. Also, the starting length under the lossy condition (*σ* = 2 × 10^7^ S/m) shown in Figure 4b is about 7.79 mm.

### 4.4. Starting Length by PIC Simulation

**Figure 5:**
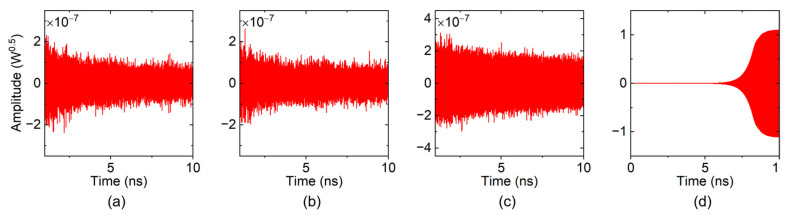
Time-domain signals from the “input port” of the loss-free sample TWT: (**a**) 52 cells; (**b**) 56 cells; (**c**) 60 cells; and (**d**) 62 cells.

**Figure 6:**
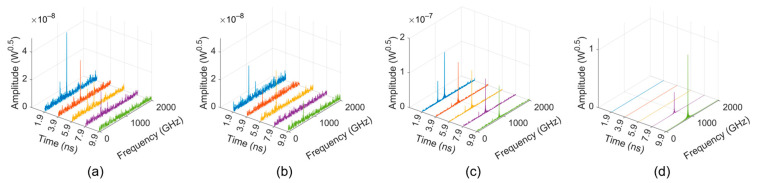
Frequency spectra of the signals from the “input port” of the loss-free sample TWT: (**a**) 52 cells; (**b**) 56 cells; (**c**) 60 cells; and (**d**) 62 cells.

In the loss-free condition, the time-domain signal output from the “input port” of the TWT is shown in Figure 5. The number of cells was gradually added until a remarkable oscillation was observed. In order to characterize the oscillation more clearly, the time-dependent spectra were computed by Fast Fourier Transform (FFT) with a 0.2 ns rectangular window, which is shown in Figure 6.

The frequency spectra of the 52- and 56-cell TWT look disordered, and their magnitudes are at the level of 10^−8^–10^−7^, as shown in Figure 6a,b. This means that there is no obvious oscillation formed in these situations. As the number of cells increased to 60, a clear peak occurred in Figure 6c, whose frequency gradually moved to 851 GHz, with a main backward-wave oscillation frequency of mode 2. However, its magnitude is rather very low (10^−7^ level), and it is stable and does not grow exponentially over time, which means oscillations are primed but not pronounced. When the number of cells is further increased to 62, a typical self-oscillation signal arises, as shown in Figure 5d. These results indicate that the starting number of cells for the loss-free sample SWS is 56–62, corresponding to a starting length of 5.77–6.39 mm.

**Figure 7:**
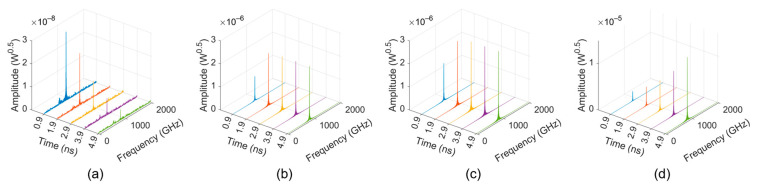
Frequency spectrum of the signals from the “input port” of the lossy sample TWT: (**a**) 60 cells; (**b**) 72 cells; (**c**) 74 cells; and (**d**) 76 cells.

Figure 7 shows the frequency spectrum results in the lossy situation, indicating that the starting number of cells for the lossy sample SWS is 72–76, corresponding to a starting length of 7.42–7.83 mm.

### 4.5. Comparison between Analytical and Simulation Solutions

This section compares the starting length for a periodic FW SWS by using two methods, which are the small-signal equation and the PIC simulation, respectively. The calculated results of the starting length for the loss-free and the lossy situations are 5.95 mm and 7.79 mm, respectively, using the small-signal equation. The computed results by the PIC simulation are in two ranges, namely 5.77–6.39 mm and 7.42–7.83 mm. The results provided by the two methods show good agreement. Hence, the simple small-signal equation would be a convenient tool for calculating the starting length in a periodic FW SWS with great accuracy.

## 5. Performance Simulation

### 5.1. Simulation of the Dispersion Characteristics

**Figure 8:**
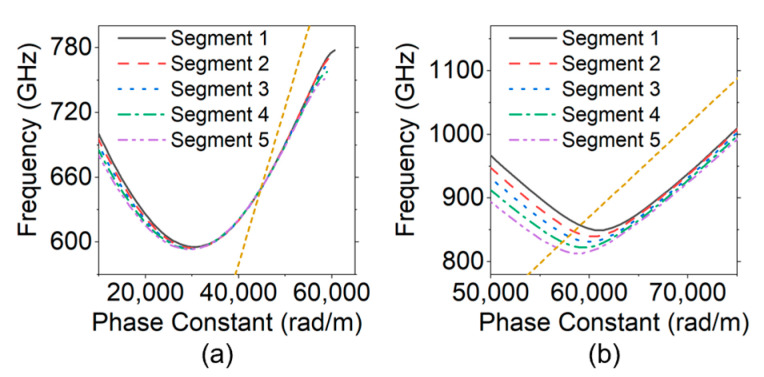
Simulation results of the dispersion characteristics of designed 650 GHz SQSST-FW SWS: (**a**) mode 1; and (**b**) mode 2.

Figure 8 shows the simulation results of the cell dispersion characteristics in different segments, where the part of Segments 6–9 was neglected due to the symmetry of the structure.

The frequency of the designed perfect-synchronous point (PSP) is 650 GHz, which can be observed in Figure 8a. By the same method in Section 4, one can find that the oscillation frequencies in Segments 2–5 are 841.8 GHz, 833.9 GHz, 824.5 GHz, and 816.2 GHz, respectively, corresponding to the main backward-wave oscillation of mode 2, as shown in Figure 8b.

### 5.2. Starting Lengths in Segments 2–5

Table 2 lists the parameters of these oscillation points, where the conductivity of the background metal is still set as *σ* = 2 × 10^7^ S/m.

**Figure 9:**
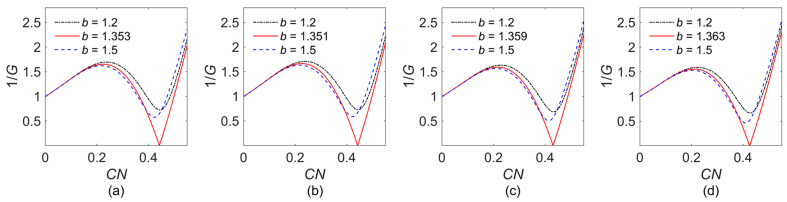
Relations of 1/*G* versus *CN*: (**a**) Segment 2; (**b**) Segment 3; (**c**) Segment 4; and (**d**) Segment 5.

The diagrams of 1/*G* versus *CN* for Segments 2–5 can then be easily obtained by substituting the data in Table 2 into (6), as shown in Figure 9.

The calculated results in Figure 9 indicate that the starting lengths for Segments 2–5 are 7.91 mm, 7.82 mm, 7.41 mm, and 7.27 mm, respectively, corresponding to cell numbers 76, 74, 70, and 68.

### 5.3. Overall Structure Design and Transmission Characteristics

As the oscillation frequencies for Segments 1–5 are different, the potential backward-wave oscillations in them are non-coherent. So, the oscillation would not start as long as the number of cells with different dimensions in the overall SWS remains less than the starting number of cells.

Following the above principle, the number of cells in Segment 5 is designed as a conservative value of 63, and that in others are all 33.

**Figure 10:**
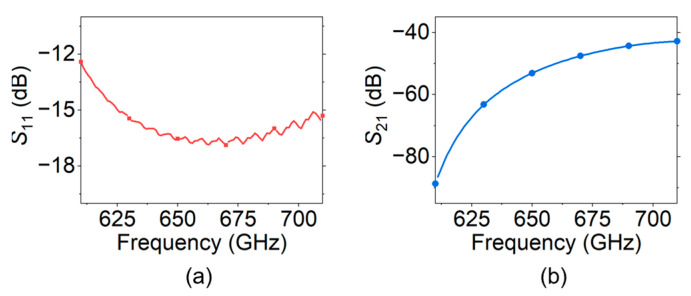
Simulation results of the characteristics of the 650 GHz SQSST-FW SWS: (**a**) *S*_11_; and (**b**) *S*_21_.

The simulation results of the transmission characteristics of the overall SWS are shown in Figure 10, where *S*_11_ is about −15 dB to −17 dB, and *S*_21_ is about −45 dB to −65 dB in the frequency range of 630 GHz–670 GHz.

### 5.4. PIC Simulation

**Figure 11:**
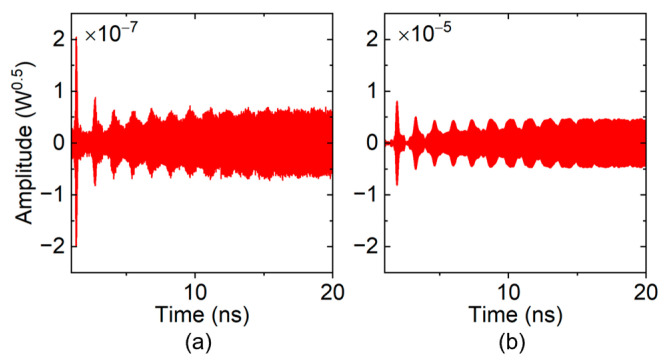
Time-domain signals from the (**a**) “input port” and (**b**) “output port” of the 650 GHz SQSST-FW TWT.

A 25.4 kV 15 mA ideal electron beam with a radius of 0.035 mm was then applied to the PIC simulation. At first, a zero-drive simulation was performed to evaluate the oscillation state in the designed 650 GHz SQSST-FW TWT. Figure 11 shows the time-domain output signals from the two ports of the TWT.

**Figure 12:**
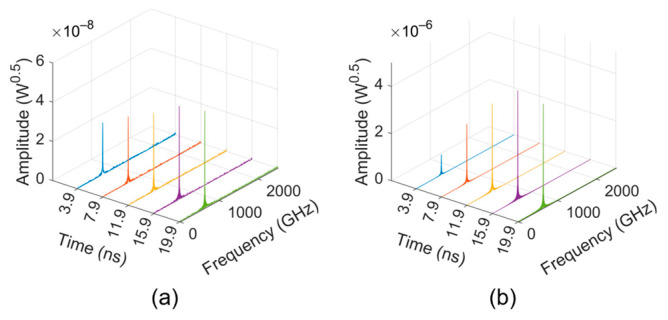
Frequency spectrum of the (**a**) “input port” and (**b**) “output port” signals of the 650 GHz SQSST-FW TWT.

The simulation results in Figure 11 indicate that the powers of the self-excited signals from the two ports of the TWT are quite low, whereas the power output from the “output port” is at the pW level and that from the “input port” is even at the fW level. The insets in Figure 11a,b show that the self-excited signals have no upward trend. The frequency spectra of these two signals are shown in Figure 12, in which one can find that the peak frequency is very close to 650 GHz, the frequency of the PSP.

According to the above information, it is reasonable to determine the output signal from the “output port” of the TWT as a tiny reflection oscillation, and the output signal from the “input port” as its reflection. The reason for such a tiny oscillation is that the phase velocity of the forward wave of 650 GHz, the frequency of the PSP, is constant along the *z*-axis, and thus the SWS has no function of suppressing reflection oscillation of this frequency.

However, there is no positive energy feedback loop for the reflection oscillation in the tube, and the steady oscillation power is only at the pW level. So, the effect of this tiny oscillation on the performance of the tube can be neglected.

**Figure 13:**
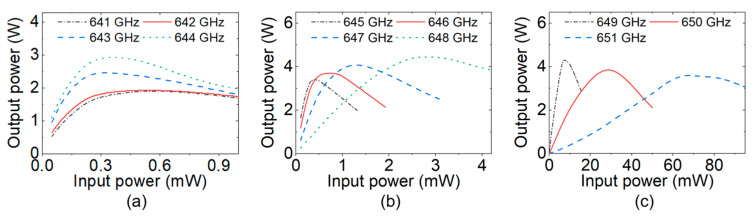
PIC simulation results of the drive curves of the 650 GHz SQSST-FW TWT: (**a**) 641 GHz–644 GHz; (**b**) 645 GHz–648 GHz; and (**c**) 649 GHz–651 GHz.

**Figure 14:**
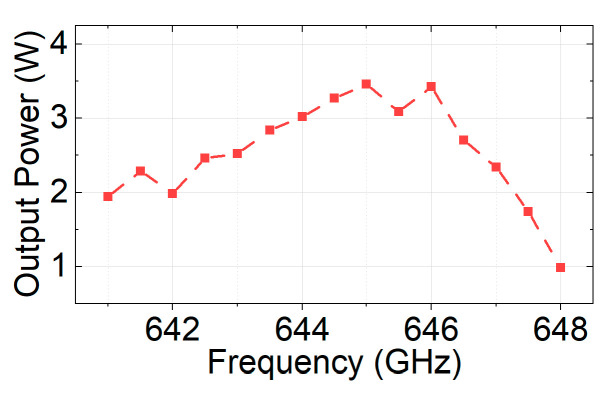
PIC simulation results of the equal-drive output powers at different frequencies.

Figure 13 shows the drive curves in the frequency range of 641 GHz–651 GHz with a step of 1 GHz, and Figure 14 shows the output powers at different frequencies with a fixed 0.4 mW input power.

The PIC simulation results indicate that the designed SQSST-FW TWT has a saturated output power of 3.46 W when the input power is 0.43 mW at 645 GHz, corresponding to a gain of 39.1 dB and an electronic efficiency of 0.91%. In addition, the maximum output power can reach 4.46 W when the input power is 2.8 mW at 648 GHz. The gain would be reduced to 32 dB, but the electronic efficiency would increase to 1.17%. In addition, the 3 dB bandwidth with a 0.4 mW input power is about 6.5 GHz.

## 6. Discussion

In Section 3 and Section 4, the backward-wave oscillation starting length for the sample periodic FW SWS is analyzed by 1-D linear theory for BWOs and successfully verified by PIC simulations. However, it has actually been conditionally simplified there, including ignoring the impact of the plasma frequency reduction factor and adopting the simplified formula for the space charge parameter ($QC=\omega_{p}^{2}/\omega^{2}/C^{2}/4$, where $\omega_{p}$ is the plasma frequency). The condition is that both *C* and *QC* are relatively low, which can be satisfied in most terahertz FW TWTs. For the TWTs with high *C* or high *QC*, the calculation results may need to be modified.

The designed 650 GHz SQSST-FW TWT performs well in gain, output power and electronic efficiency but shows disadvantages in some other respects. One disadvantage is its relatively narrow 3 dB bandwidth, about 1%, and another is the high requirement for machining accuracy. Further detailed studies need to be conducted to explore its optimization potential.

## 7. Conclusions

This paper presents a novel SQSST-FW SWS based on the QSR and a design method for it by the combination of the 1-D linear theory for BWOs and numerical simulations, which is illuminated by a design example for 650 GHz TWT applications. The designed SQSST-FW SWS consists of nine segments, which are symmetrical about the fifth segment. Following the principle that the cell number in every segment did not exceed the oscillation-starting cell number, the finally designed 650 GHz SQSST-FW TWT reached a maximum saturated gain of 39.1 dB without the help of attenuators or severs in PIC simulation. It also performs well in output power and electronic efficiency. The simulation results verify the validity of the proposed concept and method and show the great potential of the SQSST-FW SWS for applications in terahertz high-gain high-power TWTs.

## Figures and Tables

**Table 1:** Cell dimensions in different segments.

Symbol	Value (mm)	Symbol	Value (mm)
*p*_1_	0.103	*h*_1_	0.105
*p*_2_	0.10403	*h*_2_	0.109
*p*_3_	0.10478	*h*_3_	0.113
*p*_4_	0.10578	*h*_4_	0.118
*p*_5_	0.10682	*h*_5_	0.123

**Table 2:** Oscillation-point parameters in Segments 2–5.

Segment	Frequency (GHz)	Interaction Impedance (Ω)	Loss Parameter *d*
2	841.8	1.5	0.511
3	833.9	1.6	0.516
4	824.5	1.8	0.489
5	816.2	1.9	0.473

## Data Availability

Data are contained within the article.
